# Skin infiltrating T-cell profile of drug reaction with eosinophilia and systemic symptoms (DRESS) reactions among HIV-infected patients

**DOI:** 10.3389/fmed.2023.1118527

**Published:** 2023-05-05

**Authors:** Tafadzwa Chimbetete, Phuti Choshi, Sarah Pedretti, Mireille Porter, Riyaadh Roberts, Rannakoe Lehloenya, Jonathan Peter

**Affiliations:** ^1^Division of Allergy and Clinical Immunology, Department of Medicine, University of Cape Town, Cape Town, South Africa; ^2^Allergy and Immunology Unit, University of Cape Town Lung Institute, Cape Town, South Africa; ^3^Division of Anatomical Pathology, Department of Pathology, University of Cape Town, Cape Town, South Africa; ^4^Division of Dermatology, Department of Medicine, University of Cape Town, Cape Town, South Africa; ^5^Combined Drug Allergy Clinic, Groote Schuur Hospital, Cape Town, South Africa

**Keywords:** drug reaction with eosinophilia and systemic symptoms, human immunodeficiency virus, immunohistochemistry, severe cutaneous adverse reaction, T-cell, first-line tuberculosis drug

## Abstract

**Introduction:**

Drug Reaction with Eosinophilia Systemic Symptoms (DRESS) is more common in persons living with HIV (PLHIV), and first-line anti-TB drugs (FLTDs) and cotrimoxazole are the commonest offending drugs. Limited data is available on the skin infiltrating T-cell profile among DRESS patients with systemic CD4 T-cell depletion associated with HIV.

**Materials and methods:**

HIV cases with validated DRESS phenotypes (possible, probable, or definite) and confirmed reactions to either one or multiple FLTDs and/or cotrimoxazole were chosen (*n* = 14). These cases were matched against controls of HIV-negative patients who developed DRESS (*n* = 5). Immunohistochemistry assays were carried out with the following antibodies: CD3, CD4, CD8, CD45RO and FoxP3. Positive cells were normalized to the number of CD3+ cells present.

**Results:**

Skin infiltrating T-cells were mainly found in the dermis. Dermal and epidermal CD4+ T-cells (and CD4+/CD8+ ratios) were lower in HIV-positive vs. negative DRESS; *p* < 0.001 and *p* = 0.004, respectively; without correlation to whole blood CD4 cell counts. In contrast, no difference in dermal CD4+FoxP3+ T-cells was found in HIV-positive vs. negative DRESS, median (IQR) CD4+FoxP3+ T-cells: [10 (0–30) cells/mm^2^ vs. 4 (3–8) cells/mm^2^, *p* = 0.325]. HIV-positive DRESS patients reacting to more than one drug had no difference in CD8+ T-cell infiltrates, but higher epidermal and dermal CD4+FoxP3+ T-cell infiltrates compared to single drug reactors.

**Conclusion:**

DRESS, irrespective of HIV status, was associated with an increased skin infiltration of CD8+ T-cells, while CD4+ T-cells were lower in HIV-positive DRESS compared to HIV-negative DRESS skin. While inter-individual variation was high, the frequency of dermal CD4+FoxP3+ T-cells was higher in HIV-positive DRESS cases reacting to more than one drug. Further research is warranted to understand the clinical impact of these changes.

## Introduction

Severe cutaneous adverse drug reactions (SCARs) are off-target T-cell-mediated cutaneous adverse drug reactions (CADR) that are life-threatening and are associated with high morbidity, mortality, and healthcare costs (1, 2). Drug reaction with eosinophilia and systemic symptoms (DRESS) is one of the commonest SCARs globally with estimated prevalence of 4/10000 in the general population (3). Persons living with HIV (PLHIV) may have up to a 100-fold increased risk of SCAR compared to HIV-negative persons, and 1 in 10 PLHIV experiences a treatment-limiting ADRs during multi-drug resistant tuberculosis (TB) regimens (1, 4). The high prevalence of comorbid TB in HIV endemic settings, means first-line anti-TB drugs (FLTDs) and cotrimoxazole (used for *Pneumocystis jirovecii* prophylaxis) are the commonest offending agents causing DRESS in the South African setting (3, 5). PLHIV experiencing treatment-limiting FLTD/cotrimoxazole-associated DRESS are a vulnerable population with considerable complexity including: high TB-HIV disease related mortality; polypharmacy; intercurrent infectious and non-infectious co-morbidities; and prolonged hospitalization (5). There is urgent need to understand DRESS in the context of PLHIV to develop predictive biomarkers and improved treatments.

The immunopathogenesis of DRESS is evolving. Current understanding of DRESS pathogenesis includes drug specific immune responses and reactivation of human herpes viruses (HHVs). Both CD4+ and CD8+ effector T-cells are thought to be pathogenic mediators of DRESS, and enhanced dermal lymphatic infiltration of lymphocytes and eosinophils, together with an increased secretion of inflammatory cytokines including tumor necrosis alpha and interferon gamma (IFN-γ), are the main characteristics of DRESS. Reactivation of HHVs including HHV6, Epstein–Barr virus and cytomegalovirus often occurs during the acute and recovery period of DRESS, although the process by which viral reactivation contributes to DRESS pathogenesis is currently unclear (3, 6). There is even more limited data from DRESS in PLHIV; with several immunological and physiological changes through the life-cycle of HIV that could alter typical DRESS pathology and pathogenesis (5). CD4+ T-cell depletion is the hallmark of advanced HIV-related immunosuppression, but additional immune dysregulation such as expansion of effector memory T-cells and possible depletion of T-cell regulatory mechanisms have been proposed to play a key role in driving the increased DRESS prevalence in PLHIV (5). However, detailed immunohistological study of site-of-disease samples has been lacking, with only one study in toxic epidermal necrolysis (TEN) reported (7). Thus, this study sought to characterize the immunophenotype of DRESS among PLHIV co-infected with TB, with challenge reactions to one or more FLTD and/or cotrimoxazole. We compared immunohistology of HIV-positive and negative FLTD/cotrimoxazole associated DRESS patients, with a hypothesis that immune dysregulation may cause depletion of protective CD4+FoxP3+ T-cells and increased infiltration of CD8+ T-cells in the skin of PLHIV. We furthermore examined for immunohistological features that may predict patients likely to have clinical reactivity to more than one drug during sequential drug challenge (SDC) to FLTDs and/or cotrimoxazole.

## Materials and methods

### Patient selection, case validation, and drug causality assessment

The Immune-Mediated Adverse Drug Reactions (IMARI) Registry and Biorepository is the first multidisciplinary drug allergy clinic to be established on the African continent and was developed through the Groote Schuur Hospital Combined Drug Allergy Clinic (Cape Town, South Africa) (8). For this study, HIV-positive and negative patients in the IMARI Registry and Biorepository admitted with FLTD and/or cotrimoxazole-associated DRESS were selected. DRESS cases were validated by two expert dermatologists as “probable” or “definite” based on the RegiSCAR scoring system (9). Inclusion of “possible” cases of DRESS was by expert opinion.

Drug causality assessment reports were carried out for all possible offending drugs. This included Naranjo probability scores for DRESS suggesting a “possible,” “probable” or “definite” reaction to FLTDs or cotrimoxazole (10). Additionally, adjunctive enzyme-linked immunospot assay (ELISpot) data were extracted, where possible to aid confirmation of most likely causative drug and phenotype. IFN-γ ELISpot assays were run on patient peripheral blood mononuclear cells (PBMCs) collected at the acute stage of DRESS, before SDC, and or on positive challenge reaction to FLTDs. PMBCs were stimulated using optimized stimulating drug concentrations and positive ELISpot results were considered to be ≥50 spot forming units (SFU) per million cells (11, 12).

### Sampling of the skin

Skin biopsies were performed during the acute phase of DRESS, approximately between 1 to 20  days from the onset of clinical symptoms. Patients sampled were not on systemic corticosteroids or other immunosuppressive treatments at the time of skin biopsy. A 4 mm skin biopsy taken from lesional skin with maximal erythema and infiltration was sent to the National Health Laboratory Service (NHLS) for routine histopathological evaluation. Cases both clinically and histopathologically compatible with FLTD/cotrimoxazole-induced DRESS were included in this study. Four millimeters punch biopsies matched for HIV status, were obtained at a morphologically normal site from discarded healthy skin collected from surgical procedures, i.e., breast reduction or mastectomies to serve as normal controls.

### Immunohistochemistry staining of T-cell infiltrates

Skin biopsies of those meeting inclusion criteria were fixed in 10% formalin, embedded in paraffin, and were cut into sections of 10 μm thickness. Sections were blocked using 1% bovine serum albumin in phosphate buffer saline (PBS) for 1 h at room temperature. Immunohistochemistry (IHC) staining was performed using primary antibodies (Supplementary Table S1) monoclonal rabbit anti-human CD3 (clone SP7, Abcam), monoclonal rabbit anti-human CD4 (clone EPR6855, Abcam), polyclonal rabbit anti-human CD8 (#Ab4055, Abcam), and monoclonal mouse anti-human CD45RO (clone UCHL1, Biolegend). Horseradish peroxidase conjugated (HRP) human anti-rabbit (#K4003, DAKO) and HRP human anti-mouse (#K4001, DAKO) were used as secondary antibodies. Slides were washed in 1X PBS in between blocking and antibody incubation. Sections were incubated with 3,3’-Diaminobenzidine (DAB) chromogen (#K3468, DAKO) and counterstained with hematoxylin to allow visualization of nuclei. Imaging and quantification was performed using the ZEISS Primo Star Light Microscope (Zeiss, Germany). Briefly, for each antibody staining, a center section of biopsy specimen was stained for CD3 as a marker for lymphocytes. Adjacent sections were stained for CD4, CD8 or CD45RO antibodies. An area of high density CD3+ staining was identified at low-power magnification (10X) on the section for both epidermal and dermal compartments and captured. The selected area of high-density staining was retraced at high-power magnification (40X) in the corresponding set of adjacent CD4+, CD8+ or CD45RO+ staining, and images were captured. The total number of positive cells in interrelated images, normalized to CD3+ cells present were manually counted and averaged over three high-powered successive fields. Epidermal and dermal compartments were quantified separately, and cells appearing at the dermoepidermal junction were considered to be in the epidermis (7, 13).

### Immunofluorescence staining of CD4+FoxP3+T-cells

After blocking of non-specific binding in 1% bovine serum albumin, sections were immunostained using primary antibodies (Supplementary Table S1) monoclonal rat anti-human CD3 (clone CD3-12, Abcam), monoclonal rabbit anti-human CD4 (clone EPR6855, Abcam), and monoclonal mouse anti-human FoxP3 (clone 236A/E7). Alexa Fluor 488-conjugated (A488) donkey anti-rat, A647-conjugated donkey anti-mouse, and Cy3-conjugated donkey anti-rabbit were used as secondary antibodies. Sections were counterstained with 4′,6-diamidino-2-phenylindole (DAPI) to allow visualization of nuclei, washed and incubated with 0.1% Sudan Black B in 70% ethanol to reduce autofluorescence background staining. Imaging was performed using a Zeiss LSM880 confocal scanning microscope (Zeiss, Germany) and positive cells were quantified as per Gray et al. (2020) (14). Briefly, for each section, tilescan images (stitched panels) spanning the tissue to include the epidermis and dermis were captured at 60X magnification. As the majority of CD4+FoxP3+ T-cells infiltrated the superficial to mid dermis, we set our tilescans to include at least three X60 images spanning the dermis, and *n* X60 images in the epidermis depending on the thickness. A minimum of 4 tilescan images along the skin section were captured for each section/patient. CD3+CD4+FoxP3+ T-cells in both dermal and epidermal compartments of each tilescan were identified and manually counted. The total number of dermal or epidermal CD4+FoxP3+ T-cells in each captured tilescan image was divided by the area of each respective compartment to calculate density (cells/mm^2^). This density of positive cells was averaged over the total number of images quantified for each section (14, 15).

### Statistical analysis

Statistical analysis was performed using R Studio version 4.0.5 and SPPS Statistics 27. Data were non-parametric and so median (interquartile ranges) are presented and Wilcoxon rank sum testing statistical testing used; significance was set at *p* value <0.05. Multivariate linear regression was used to assess any correlation between the density of CD4+FoxP3+ T-cells and clinical characteristics; *p* values <0.05 were considered statistically significant. No correction for multiple comparisons were performed.

## Results

### DRESS cases and drug causality

HIV, TB, and DRESS summary clinical and laboratory data, stratified by HIV status at baseline, is detailed in Table 1, and further detailed clinical description of reactions at baseline as well as SDC reactivations, ELISpot results and Naranjo drug causality scoring for each patient is shown in Supplementary Table S2 (HIV-positive) and S3 (HIV-negative). Most HIV-positive DRESS cases were female, with a median age and CD4 cell count (IQR) of 40 (30–45) years and 112 (66–288) cells/mm^3^, respectively. The drug latency period and number of days between onset of clinical symptoms to sampling was 26 (12–31) days and 9 (5–13) days respectively, with no differences between HIV-positive and negative DRESS cases. Patient 15 was sampled 73 days after onset of symptoms; hence he was excluded from the comparative analyses of patients sampled in the acute phase of DRESS (~ 1 to 20 days from symptoms onset) but rather detailed as a unique case report. During SDC of HIV-positive DRESS cases, 10 were clinically reacting to a single drug (single drug reactors), while two cases had a positive challenge reaction to two or more drugs (multiple drug reactors). Among HIV-negative patients, three cases were reacting to a single drug, while two were multiple drug reactors. The clinical and laboratory data of HIV-positive and negative normal skin controls included in the study are highlighted in Supplementary Table S4.

**Table 1:** Summary of clinical and laboratory data for HIV-positive versus negative DRESS patients.

Variables	All DRESS patients (*n* = 19)		
	HIV-positive (*n* = 14)	HIV-negative (*n* = 5)	*p* value
Age in years, median (IQR)	40 (30–45)	46 (15–52)	0.75
Sex: Female, *n* (%)	11/14 (79)	3/5 (60)	-
Male, *n* (%)	3/14 (21)	2/5 (40)	-
CD4 cell count, median (IQR)	112 (66–288)	-	-
On antiretroviral therapy at time of DRESS, *n* (%)	7/14 (50)	-	-
Validated phenotype, *n* (%): Definite	7/14 (50)	3/5 (60)	-
Probable	4/14 (29)	2/5 (40)	-
Possible	3/14 (21)	0	-
Type of tuberculosis, *n* (%): Pulmonary	10/14 (71)	1/5 (17)	-
Disseminated	3/14 (21)	0	-
No active TB	1/14 (7)	4/5 (83)	-
Drug latency period, median days (IQR)	26 (12–31)	34 (32–36)	0.09
Days from symptoms onset to biopsy, median (IQR)	9 (5–13)	6 (3–8)	0.26
Percentage body surface area (BSA) skin rash, median (IQR)	65 (56–74)	40 (40–40)	0.32
Liver function tests (reference values): *n* (%)			
AST (15–40 U/l): Normal	2/14 (14)	1/5 (20)	1
2–5x ULN	6/14 (43)	2/5 (40)	1
>5x ULN	1/14 (7)	2/5 (40)	0.15
ALP (53–128 U/l): Normal	10/14 (71)	2/5 (40)	0.3
2–5x ULN	2/14 (14)	2/5 (40)	0.27
>5x ULN	1/14 (7)	1/5 (20)	0.47
ALT (10–40 U/l): Normal	5/14 (36)	0	0.26
2–5x ULN	2/14 (14)	2/5 (40)	0.27
>5x ULN	3/14 (21)	2/5 (40)	0.57
GGT (<68 U/l): Normal	5/14 (36)	0	0.26
2–5x ULN	4/14 (29)	1/5 (20)	1
>5x ULN	2/14 (14)	2/5 (40)	0.27
Eosinophil count (reference value: 0.0–0.4 ×10^9^/L)	0.63 (0.2–1.42)	1.36 (1.36–2.55)	0.13

ALT, alanine transaminase; ALP, alkaline phosphatase; AST, aspartate transaminase; BSA, body surface area; DRESS, drug reaction with eosinophilia and systemic symptoms; GGT, gamma-glutamyl transferase; HIV, human immunodeficiency syndrome; IQR, interquartile range; TB, tuberculosis; ULN, upper limit of normal.

### Basic histopathological similarities in HIV-positive and negative DRESS compared to normal skin

**Figure 1:**
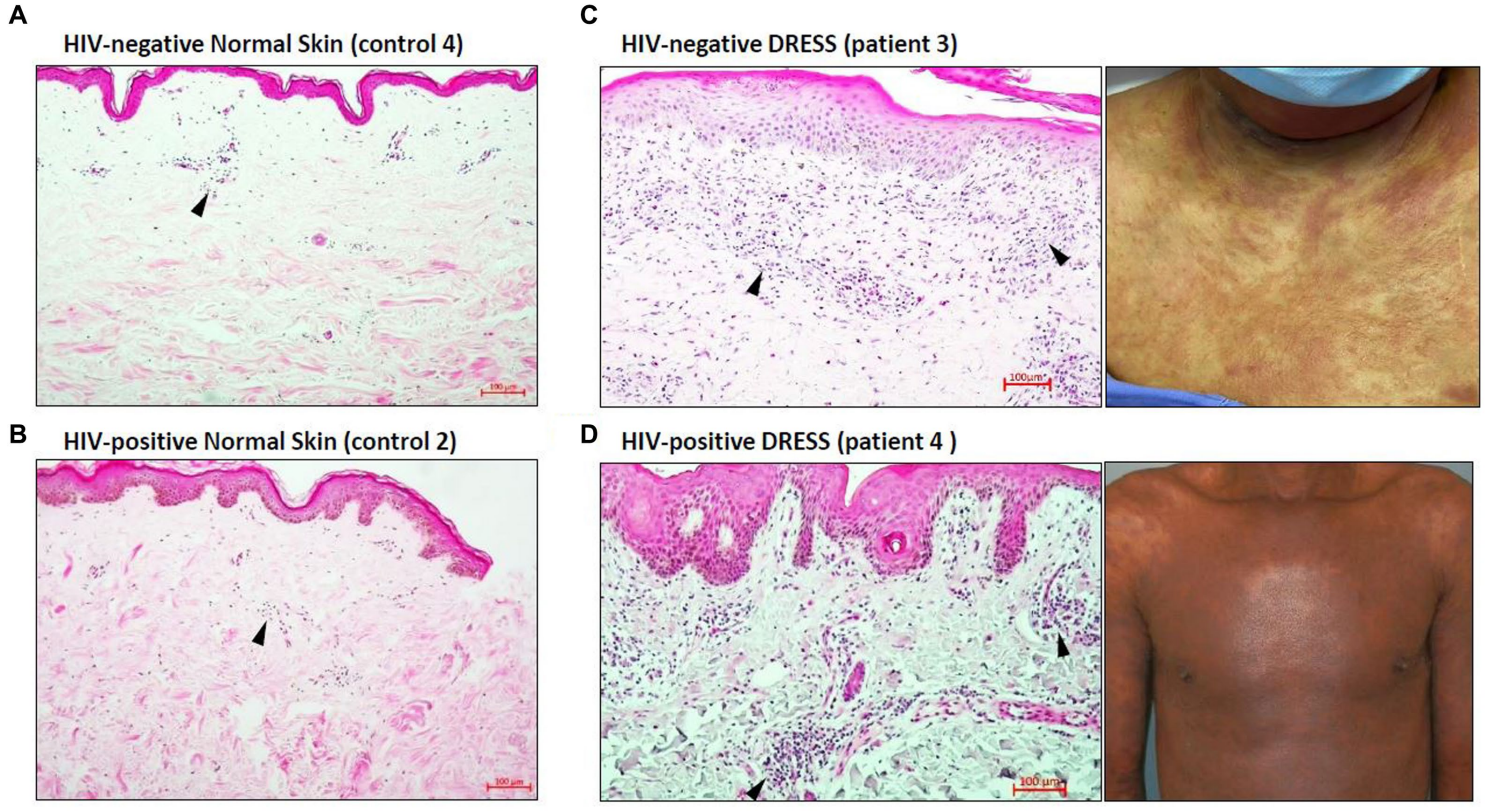
Distribution of inflammatory infiltrates in the skin of HIV-negative and positive normal skin versus HIV-negative and positive DRESS skin with matched macroscopic images. Increased dermal and epidermal infiltrates can be noted among HIV-negative **(C)** and positive **(D)** DRESS skin compared to HIV-negative **(A)** and positive **(B)** normal skin. Similarly, secondary histopathological changes can be seen in both HIV-negative and positive DRESS skin, and none observed in normal skin. Black arrowheads denote inflammatory infiltrates. DRESS, drug reaction with eosinophilia and systemic symptoms; HIV, human immunodeficiency virus; NS – normal skin. Scale bars = 100 μm.

Histopathological features of diagnostic or prognostic importance were identified and the histopathologic diagnosis was confirmed by a Consultant Anatomical Pathologist (RR). Figure 1 is a hematoxylin and eosin (H&E) staining showing the overall distribution of inflammatory infiltrates in the skin of representative HIV-positive and negative DRESS patients compared to normal skin. As expected, a higher infiltration of cells can be noted in the skin undergoing a DRESS reaction, whereas very little activity is noted in normal skin. The absence of any secondary histopathological changes characteristic of DRESS that develop in epidermal or dermal compartments can also be noted in normal skin. Among HIV-positive and negative DRESS patients, common histopathological changes observed included epidermal spongiosis (86% HIV-positive, 75% HIV-negative DRESS), basal vacuolar degeneration (53, 50%), a dermal infiltrate of predominantly lymphocytes and eosinophils (57, 50%) and pigmentary incontinence (47, 50%). Due to the small sample size, no significant differences were observed in histopathological changes among HIV-positive and negative DRESS patients, however a trend was observed in the following parameters: focal interface dermatitis (46% HIV-positive, 100% HIV-negative; *p* = 0.10), perivascular dermal inflammation (53, 100%; *p* = 0.25), apoptotic keratinocytes (80, 25%; *p* = 0.07), and epidermal exocytosis (6, 50%; *p* = 0.09).

### Skin infiltrating T-cells differ among HIV-positive and negative DRESS patients

**Figure 2:**
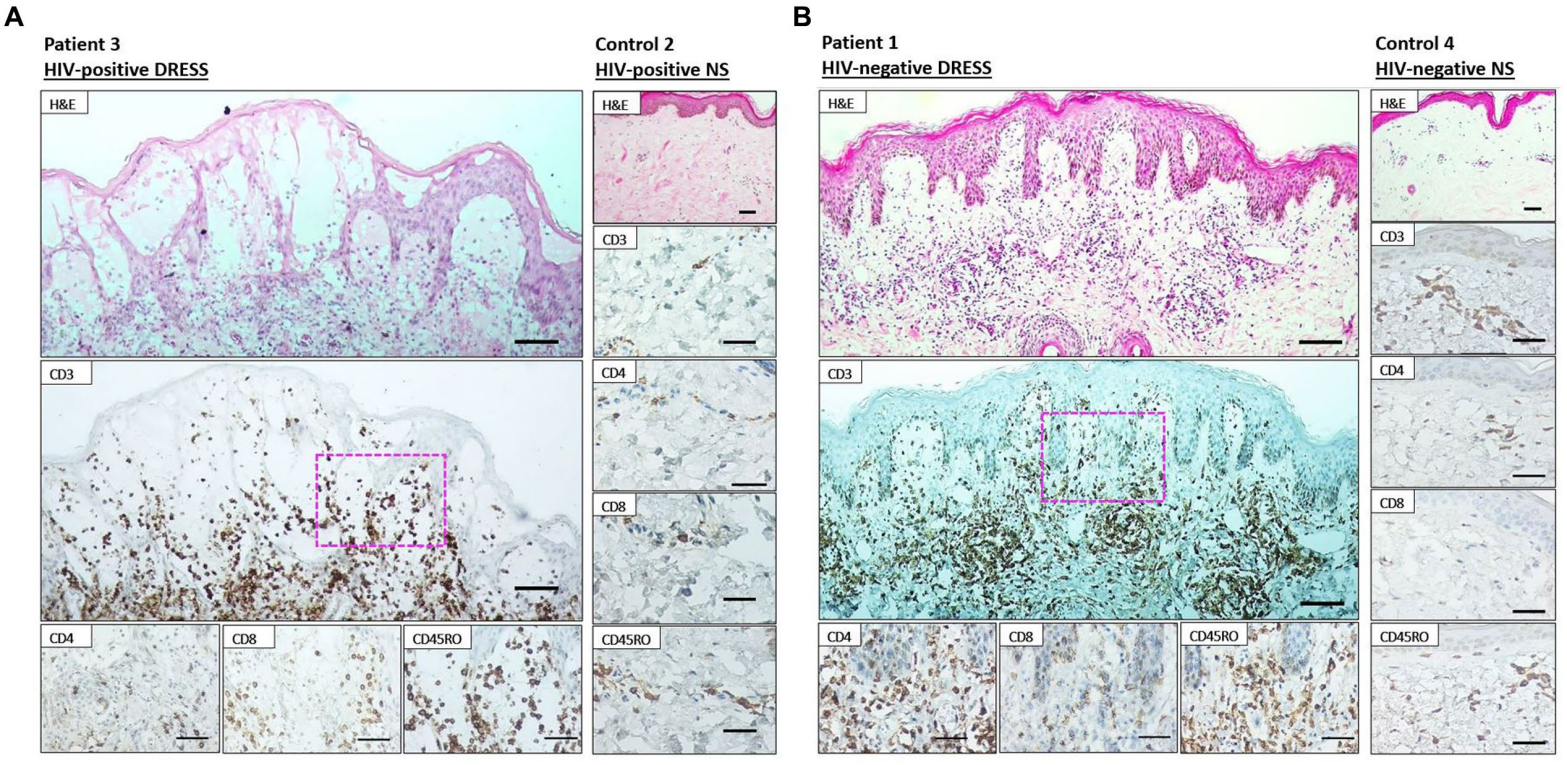
Immunohistochemistry staining of lesional skin among representative HIV-positive and negative DRESS patients and normal skin. Labeling of HIV-positive **(A)** and negative **(B)** DRESS and normal skin with rabbit anti-CD3, rabbit anti-CD4, rabbit ant-CD8, and mouse anti-CD45RO. H&E images scale bars = 50 μm. IHC images scale bars = 50 μm.

As revealed in Figure 1, a low distribution of inflammatory infiltrates was noted among HIV-positive and negative normal skin. Infiltration of T-cells within the dermis and epidermis of HIV infected and uninfected DRESS patients was increased when compared to normal skin (Supplementary Table S5). Figure 2 shows the distribution of T-cells in representative HIV-positive and negative DRESS cases and normal skin. Table 2 summarizes the quantification of T-cell infiltrates among HIV-positive and negative DRESS cases. CD3+ T-cell infiltrates were significantly increased in the dermis of HIV-negative DRESS patients (*p* = 0.03). There was a significant decrease observed in the number of dermal and epidermal CD4+ T-cells among HIV-positive DRESS patients (*p* < 0.001 and *p* = 0.004, respectively). This CD4+ depletion was associated with a significant decrease in the ratio of dermal and epidermal CD4+/CD8+ T-cells among HIV-positive DRESS (*p* < 0.001 and *p* = 0.001, respectively). No significant differences were observed in dermal and epidermal numbers of CD8+ T-cells among HIV-positive and negative DRESS patients. Using CD45RO as the memory marker, there was a trend toward increased dermal CD45RO+ T-cells observed among HIV-negative compared to HIV-positive DRESS patients, although not statistically significant (*p* = 0.22). However, double immunofluorescent (IF) immunostaining of CD4+/CD8+ with the memory marker CD45RO+ revealed no differences among HIV-positive and negative DRESS patients in both dermal and epidermal compartments (data not shown). The immunohistological phenotypes between HIV-positive DRESS patients reacting to a single drug were compared to those reacting to multiple unrelated medications. There was a trend toward increased dermal infiltration of CD4+ T-cells among multiple drug reactors compared to single drug reactors (Supplementary Figure S1A). However, no difference in the peripheral blood CD4 cell counts were noted among HIV-positive DRESS cases reacting to single versus multiple drugs, mean CD4 count (IQR) 87 (53–282) cells/mm^3^ and 104 (85–122) cells/mm^3^ respectively, *p* = 1.00.

**Table 2:** Quantification of dermal and epidermal T-cell infiltrates among HIV-positive and negative DRESS patients.

DRESS		Average number of positive cells per high-powered field		
		HIV-positive (*n* = 14)	HIV-negative (*n* = 5)	*p* value
Epidermis:	CD3	4 (2–10)	10 (8–11)	0.09
	CD4	**1 (0–2)**	**6 (5–8)**	**0.004**
	CD8	2 (1–5)	6 (3–7)	0.23
	CD45RO	2 (1–8)	4 (3–7)	0.35
	CD4/CD8	**0.3 (0.0–0.5)**	**1.7 (1.2–2.8)**	**0.001**
				
Dermis:	CD3	**53 (45–62)**	**81 (63–106)**	**0.03**
	CD4	**19 (14–24)**	**64 (46–99)**	**<0.001**
	CD8	37 (30–51)	50 (27–87)	0.60
	CD45RO	42 (33–50)	55 (36.8–81.0)	0.22
	CD4/CD8	**0.4 (0.3–0.6)**	**1.8 (1.2–2.2)**	**<0.001**

Cell counts are median (IQR). The Wilcoxon rank sum test was used to determine statistical significance between groups. *p* values < 0.05 were considered significant, recorded in bold.

### No difference in dermal CD4+FoxP3+ T-cells between HIV-positive compared to negative DRESS

**Figure 3:**
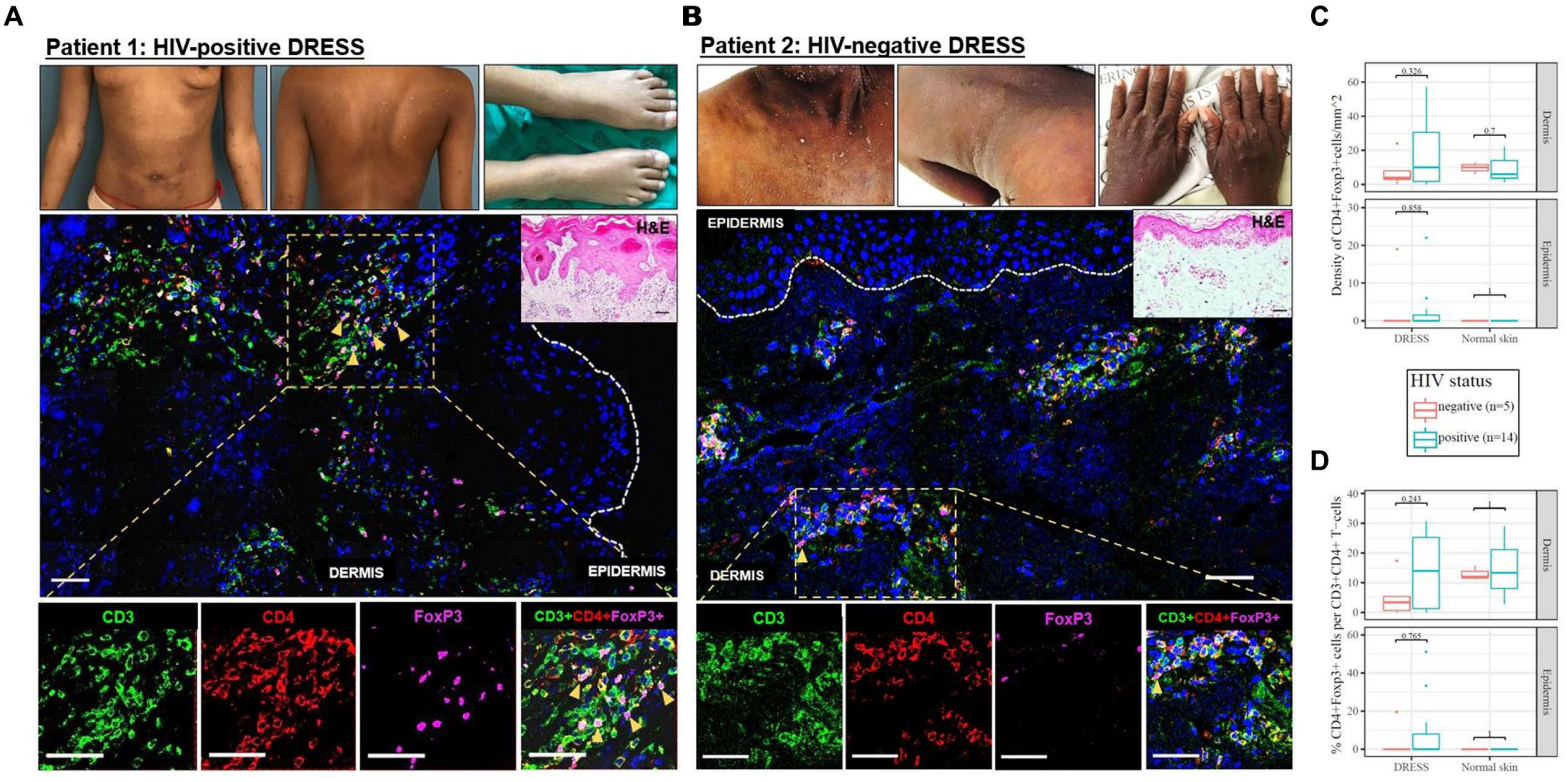
Skin CD4+FoxP3+ T-cells among representative HIV-positive and negative DRESS cases. CD4+FoxP3+ T-cell distribution in HIV-positive DRESS **(A)** and HIV-negative DRESS skin **(B)**. Lesional DRESS skin labeled with rat anti-CD3 (green), rabbit anti-CD4 (red) and mouse anti-FoxP3 (purple). Density **(C)** and frequency of CD4+FoxP3+ T-cells expressed as a percentage of CD4+ T-cells **(D)** among HIV-positive and negative DRESS and normal skin. White dotted line denotes the dermoepidermal junction. Orange arrowheads denote CD4+FoxP3+ T-cells. H&E image scale bar = 50 μm. IF images scale bar **(A)** = 100 μm, **(B)** = 50 μm.

**Figure 4:**
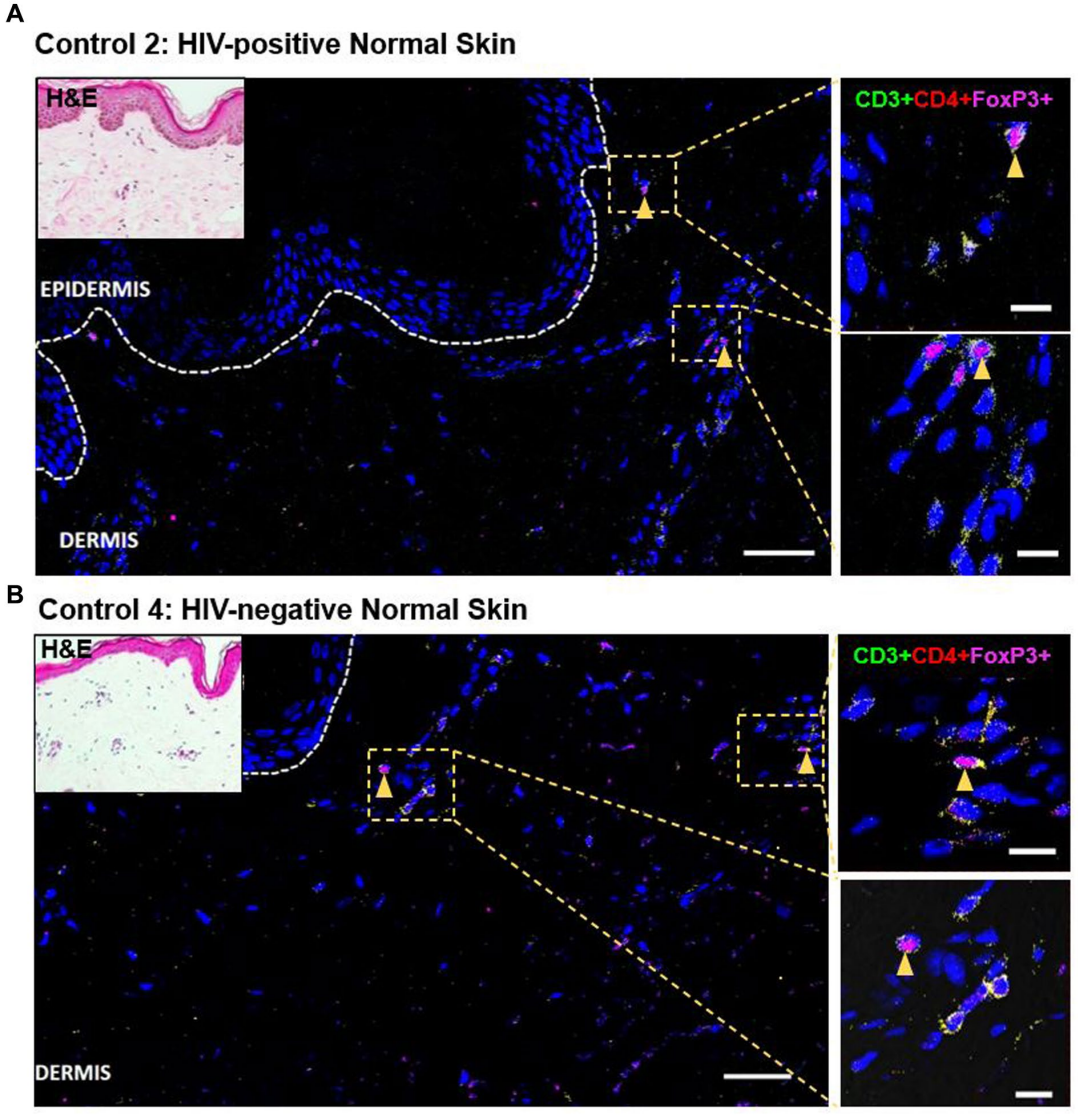
Distribution of CD4+FoxP3+ T-cells among representative HIV-positive and negative normal skin controls. A low distribution of CD4+FoxP3+ T-cells can be noted in both HIV-positive **(A)** and negative **(B)** normal skin. White dotted line denotes the dermoepidermal junction. Orange arrowheads denote CD4+FoxP3+ T-cells. Scale bar = 50 μm.

As demonstrated by IHC staining, HIV-negative DRESS was associated with a significantly increased dermal infiltration of CD4+ T-cells compared to HIV-positive. In contrast, a low and very sparse distribution of CD4+FoxP3+ T-cells was observed in the dermis, with little to none in the epidermis of HIV-negative cases (Figures 3A,B). Overall, majority of CD4+FoxP3+ T-cells, appearing in clusters of CD4+ T-cells, were infiltrating the superficial to mid-dermis, and little to none in the epidermis. There was a trend toward increased density of dermal CD4+FoxP3+ T-cells among HIV-positive DRESS compared to HIV-negative DRESS patients, *p* = 0.325 (Figure 3C). A similar trend was observed in the frequency of CD4+FoxP3+ T-cells expressed as a percentage of CD3+CD4+ T-cells present, *p* = 0.243 (Figure 3D). No significant differences in the density and frequency of dermal and epidermal CD4+FoxP3+ T-cells were observed among HIV-positive and negative normal skin (Figures 3C,D, 4).

Among HIV-positive DRESS patients reacting to multiple drugs, there was a trend toward increased density of dermal and epidermal CD4+FoxP3+ T-cells when compared to single drug reactors (Supplementary Figure S1B). We categorized the CD4+FoxP3+ T-cell counts among HIV-positive DRESS patients by the number of days from symptoms onset to skin biopsy sampling; patients sampled ≤7 days versus >7 days from the onset of clinical symptoms, and no difference in the CD4+FoxP3+ T-cell counts was observed (data not shown).

### Increased dermal and epidermal CD4+FoxP3+ T-cell infiltration in a case of DRESS with a longer delay between symptoms onset to biopsy

**Figure 5:**
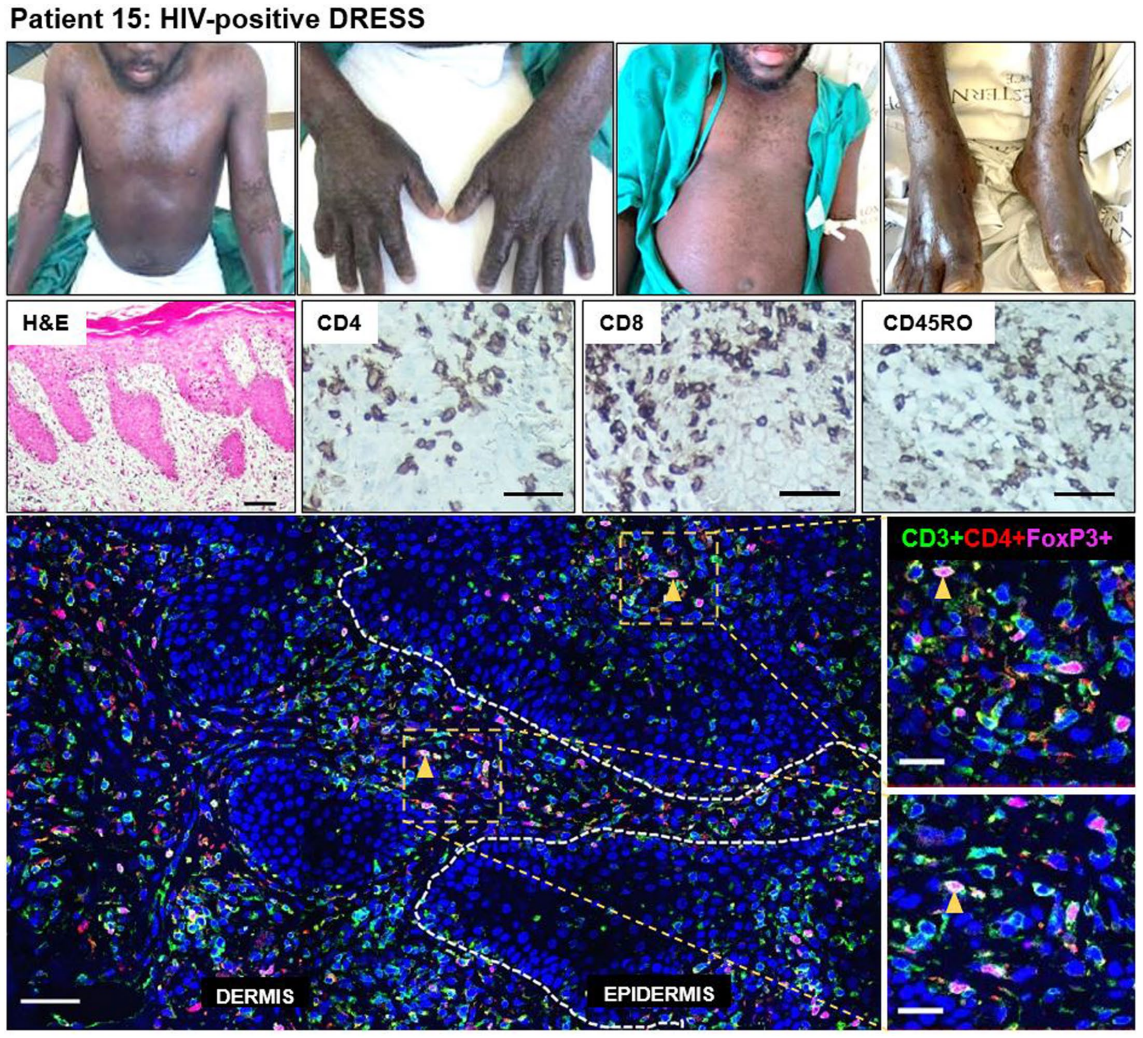
Increased skin infiltrating CD4+FoxP3+ T-cells in patient 15, HIV-positive DRESS patient, sampled 73 days from onset of clinical symptoms. A dense infiltration of CD8+ and CD45RO+ T-cells can be noted, and to a lesser extent CD4+ T-cells. The skin is infiltrated with a high number of CD4+FoxP3+ T-cells in both dermal and epidermal compartments. Orange arrowheads denote positive CD4+FoxP3+ T-cells. H&E image scale bar = 50 μm. IHC images scale bar = 50 μm. IF image scale bar = 100 μm.

A definite DRESS case (patient 15) secondary to rifampicin and isoniazid, stood out among the HIV-positive DRESS cases (disease progression timeline shown in Supplementary Figure S2). This patient was sampled 73 days after the onset of clinical symptoms, and not in the acute stage of reaction. He had been diagnosed with extrapulmonary TB [urine lipoarabinomannan positive (LAM+)] and started on rifampicin/isoniazid/pyrazinamide/ethambutol fixed dose combination (FDC). He presented with first skin symptoms characterized by an 80% body surface area (BSA) skin rash (erythema, peeling (desquamation), edema, induration), and all drugs were stopped. His most recent serum CD4 count at the time of reaction was 142 cells/mm^3^ and the patient was on antiretroviral therapy (ART). He presented with deranged liver function tests (LFTs), although the eosinophil count was normal. Histopathologic examination on H&E was consistent with a drug reaction. IHC staining revealed a very dense infiltration of CD8+ and CD45RO+ T-cells in both dermal and epidermal compartments, and CD4+ T-cells to a lesser extent (Figure 5). Despite the low infiltration of CD4+ T-cells compared to CD8+ and CD45RO+ cells, the CD4+ infiltration was higher for this patient when compared to that observed in all HIV-positive DRESS cases sampled in the acute phase of reaction. Of note though was the comparatively high infiltration of CD4+FoxP3+ T-cells, with a density of 107 and 193 cells/mm^2^ in both epidermal and dermal compartments, respectively (Figure 5).

### Association between dermal CD4+FoxP3+ and demographic and clinical characteristics

We examined for any correlations between clinical or demographic characteristics with dermal numbers of CD4+ and CD4+FoxP3+ T-cells (Supplementary Table S6). Among HIV-positive DRESS patients, there was a positive correlation between dermal CD4+FoxP3+ T-cells with the enzyme alanine transferase (ALT) (*p* = 0.01), and a negative correlation with age (*p* = 0.015). Within HIV negative DRESS patients, there was a positive correlation between dermal CD4+FoxP3+ T-cells with days from symptoms onset to sampling (*p* = 0.02), eosinophils count (*p* = 0.006), and BSA skin rash (*p* = 0.01).

An exploratory multivariate linear regression analysis was performed to determine predictors for dermal CD4+FoxP3+ T-cells among HIV-positive and negative DRESS cases (Table 3). Independent variables tested for included days from symptoms onset to biopsy, HIV status, and the BSA skin rash. No significant association was observed between dermal CD4+FoxP3+ T-cells and all the independent variables tested.

**Table 3:** Association between dermal CD4+FoxP3+ T-cells and clinical characteristics in all DRESS patients.

Independent variables	Dependent variable = dermal CD4+FoxP3+ T-cell counts(cells/mm^2^)	
	Univariate *r*^2^ (coefficient, *p* value)	Multivariate (coefficient, *p* value)
	All patients (*n* = 19)	All patients (*n* = 19) *r*^2^ = 0.102
Days from symptoms onset to sampling	0.000 (0.011, 0.965)	(−0.113, 0.68)
HIV status	0.076 (−0.275, 0.254)	(−0.26, 0.324)
% BSA skin rash	0.036 (0.189, 0.483)	(0.170, 0.536)

BSA, body surface area; HIV, human immunodeficiency virus.

## Discussion

To our knowledge, this is the first study detailing immunohistological differences among DRESS patients with and without HIV/TB coinfection; as well as examining for immunohistological features that may predict patients likely to have clinical reactivity to more than one drug during SDC to FLTDs and cotrimoxazole. Our main findings were that (i) DRESS, regardless of HIV status, was associated with an increased skin infiltration of CD8+ T-cells compared to normal skin, (ii) CD4+ T-cells were lower in the skin of HIV-positive DRESS cases compared to HIV-negative cases, (iii) the dermal infiltration of CD4+FoxP3+ T-cells was higher in HIV-positive DRESS cases reacting to more than one drug.

IHC staining of CD3, CD4, and CD8+ T-cells confirmed large numbers of these T-cells at the site of pathology in both HIV-positive and negative DRESS compared to normal skin. This supports the primary role of drug-specific lymphocytes in the immunopathology of these reactions (3, 16, 17). CD8+ T-cell infiltrates were increased in DRESS patients, irrespective of HIV status, compared to normal skin, displaying their role as key mediators of tissue damage (3). CD4+ T-cell infiltrates were reduced in the dermis and epidermis of HIV-positive DRESS when compared to HIV-negative cases, with a resultant decrease in the ratio of CD4+/CD8+ T-cells. These findings are consistent with the only other previous study by Yang et al. that characterized the composition of inflammatory infiltrates in 12 cases of TEN among HIV-positive and negative patients, reporting a decrease in CD4+ T-cell infiltrates and resultant significant increase in the ratio of CD8+/CD4+ cells in the dermis of HIV-positive patients (7). We did not find a correlation between serum and dermal CD4 T-cell counts in HIV-positive DRESS cases, indicating that although overall depletion of the circulating CD4+ T-cell pool is likely a major driver of this finding, several additional factors may impact individual CD4+ T-cell dermal infiltrates including CD4 cell activation and exhaustion status, migratory capacity/skin homing receptor expression and extent/duration of drug-induced antigenic stimulation. The relationship between the peripheral CD4 T-cell counts and likelihood of developing drug reactions has produced conflicting data. An increased likelihood of developing drug reactions with decreasing serum CD4 counts and CD4/CD8 ratio has been reported in HIV-positive individuals (18). However, early studies have identified higher CD4 cell counts (>200 cells/mm^3^) to be a predictor for SCAR including Stevens-Johnson syndrome (SJS) and drug-induced liver injury (DILI) (19–21); and there have also been studies where no association between CD4 counts and disease onset was found (22, 23). CD45RO+ T-cells predominated across CD3+ T-cell infiltrates in the dermis of DRESS patients, with no differences noted between HIV-positive and negative patients. There was, however, a trend toward increased dermal CD45RO+ T-cell expression observed in HIV-negative DRESS compared to HIV-positive cases, with the decrease among HIV-positive cases reflecting depletion in CD4+ T-cell counts. The presence of these memory cells in normal skin, particularly HIV-negative skin, shows that even in the resting unaffected/non-lesional skin, majority of resident skin T-cells have previously encountered pathogen, and are antigen-primed to allow for a swift response on secondary antigen exposure (24). There is increasing evidence that effector memory T-cells, particularly tissue resident memory (TRM) T-cells, play a role in mediating inflammatory skin disorders. Within CADR, their role in fixed drug eruptions (FDE) are the best described, where skin lesions reappear at the same skin site when patients are re-exposed to a causative drug (25, 26). In the context of SCAR, the presence of skin resident CD69+CD45RO+CD4+ and CD8+ T-cells have been reported in skin lesions of TEN and DRESS, suggesting their role in mediating disease (27, 28). It has been proposed that viral directed effector memory T-cells, can cross-react with drug-specific epitopes resulting in SCAR (16, 25, 29), therefore the specificities and role of HIV-specific TRM cells is a key future research focus.

SDC remains the gold standard in establishing drug causality and is currently the preferred method to identify offending drugs and rapidly allow the recommencement of tolerated anti-TB medications. Multiple drug hypersensitivities, neosensitisations and non-specific flare-up reactions are all described and debated in the DRESS literature (30–36). Among patients having SCAR to FLTDs and/or cotrimoxazole, reactions to multiple chemically distinct drugs is described among HIV-positive and negative cohorts (1, 36, 37). Patients reacting to multiple anti-TB drugs (and/or cotrimoxazole) during bridging and SDC pose a particular challenge for both current and future treatment of TB, where prolonged multiple drug treatment is required. Thus, an improved understanding of the risk factors of reacting to multiple drugs, and the immunopathogenesis would be very useful to define which reactions can be treated through and which need to be permanently avoided. Current hypotheses include host and drug-related factors such as: (i) true sensitization of T-cells to multiple, chemically distinct drugs, facilitated by the activated immune environment of acute DRESS, (ii) lower thresholds for non-specific flare ups, and (iii) viral reactivation of human herpesvirus (HHV) (30). In this small cohort, there were two DRESS patients that had positive drug challenge reactions to two or more structurally unrelated drugs, while 10 DRESS patients had a positive reaction to a single drug. Interestingly, our data suggests that HIV-positive patients reacting to multiple medications tended to have more dermal infiltration of CD4 T-cells and a subsequent increase in dermal and epidermal CD4+FoxP3+ T-cells compared to single drug reactors. To our knowledge there is no other reported data in literature examining T-cell infiltrates in the skin among single and multiple drug reactors, and more research is required to better detail their immunohistological phenotypes, as this may confirm these initial findings, and given the wide use of skin biopsy/histology, could provide a relatively easily accessible risk stratification tool to guide drug introduction in the post DRESS period.

Regulatory T-cells (Tregs) play a crucial role in regulating immune homeostasis and inflammation, through suppression of effector T-cells at sites of inflammation, and in a simplistic model are thereby thought to limit disease mediated by effector T-cells (38). Skin infiltration of Tregs has been studied across several inflammatory skin diseases including atopic dermatitis, psoriasis, systemic lupus erythematous and cutaneous adverse drug reactions (39–46). Surface staining markers to identify Treg cell infiltrates have included: CD4, CD25, FoxP3 and or other markers such as Helios (40–44, 47–52); but it is worth noting that functionality of Treg populations with similar surface markers can be different, meaning caution is required in direct extrapolation from number to function (39). Treg studies from SCAR are limited but increases in circulating and dermal skin infiltrating FoxP3+ Tregs in the acute phase of DRESS has been reported, with a positive correlation between dermal Treg numbers with days from rash onset found in one study (46). Takahashi et al., in a study of 11 TEN patients noted depletion of circulating CD4+CD25+ Tregs in the acute phase of TEN patients, hypothesizing that the low frequency may relate to severe epidermal damage and Treg trafficking into skin in an attempt to control immune-mediated damage (53). The increase of skin infiltrating Tregs across inflammatory diseases, including with SCAR suggests most likely that Treg infiltrations follow effector T-cell responses in a homeostatic manner. This is supported by data from Mizukawa et al. that investigated epidermal damage in evolving FDE lesions, by analyzing FDE skin lesions induced by causative drug rechallenge (54). They reported that FoxP3+ Tregs were detected predominantly in the vicinity of CD8+ T-cells after rechallenging with the causative drug, but not in resting lesions before drug rechallenge. This indicated that Treg infiltration, likely with a homeostatic suppressor function of activated effector CD8+ T-cells, follows drug-induced T-cell activation (54). Mouse data examining Treg migration into uninflamed and inflamed skin has demonstrated that molecular mechanisms controlling intraepidermal Treg migration differ considerably depending on the phase of the inflammatory response (49, 55), and although tissue-resident Tregs are immotile in the resting state, the proportion of Tregs actively migrating in the dermis is increased following innate or adaptive-induced inflammation (56). In PLHIV, the architecture of circulating immune cells is dysregulated (57). HIV-related immune dysregulation can impact the proportion and phenotype of Tregs, although the relationship is complex with several modulating factors; peripheral blood Treg numbers being shown to vary depending on the particular HIV population studies, as well as the way Tregs were characterized (58). Treg frequencies in circulation have been shown to vary according to the stage of HIV disease, ART use, and the direct effect from antigen presenting cells (APCs) activation/maturation states (47, 57–64). Studies have shown that Treg frequencies are generally increased during chronic HIV infection (58–60) and in the presence of activated and/semimature dendritic cells (DCs) in HIV (63, 64). Treg frequencies have also been shown to increase at ART onset, although normalizing with continued treatment use (57, 58, 61). In our small cohort, the majority of patients had advanced immune suppression (CD4 < 200 cells/mm^3^) but over half were established on ART, therefore making it hard to predict CD4+FoxP3+ T-cell proportions, numbers, or functional state in the context of a new DRESS. This study is the first study to examine skin infiltration of CD4+FoxP3+ T-cells in the context of DRESS patients with HIV infection. Contrary to our initial hypothesis that CD4+FoxP3+ T-cells could be depleted in the context of advanced HIV infection and poorly migrate into skin, we did not note a difference between CD4+FoxP3+ T-cell density among HIV-positive or negative DRESS patients. In fact, there was a trend to an increased number of CD4+FoxP3+ T-cells cells in HIV-positive DRESS patients, which may reach statistical significance with an increased sample size. We explored and did not find any obvious impact of confounders such as demographic and clinical characteristics to account for differences in dermal CD4+FoxP3+ T-cells between HIV-positive and negative cases. To our knowledge, there is no reported data in literature on CD4+FoxP3+ T-cells or other Tregs in circulation or skin in the context of HIV-positive DRESS, however, it may be that the continued immune activation in both HIV, TB, and DRESS drive expansion of CD4+FoxP3+ T-cell populations, and that this may be occurring in both peripheral blood and the site of disease. Mouse data suggests that exposure of hapten to naïve mice can induce an increase in the proportion of migratory Tregs, demonstrating that innate signals can induce Treg migration (56). Thus, it could be that in HIV-positive patients under skin inflammatory conditions, with innate and antigen-specific adaptive stimuli, drive an increase of CD4+FoxP3+ T-cells in the context of HIV-positive DRESS. Further work on a larger sample size is now required to confirm these findings.

The above suggested factors driving an increased CD4+FoxP3+ T-cell infiltration into skin in the context of HIV and ongoing antigenic stimulation is supported by patient 15, a definite DRESS case sampled 73 days after the onset of clinical symptoms. His disease progression timeline in Supplementary Figure S2 reveals that despite presenting with ongoing clinical symptoms suggestive of a drug reaction, there was continued attempts by the treating clinicians to treat through the grumbling reaction, and thus he had prolonged drug exposure prior to biopsy and after the development of the initial symptoms/DRESS. Thus, it is reasonable to propose that this ongoing drug exposure continued to drive infiltration of drug-specific and memory T-cells in the skin, and simultaneously CD4+FoxP3+ T-cell infiltration. Although simplistic, the relative dominant CD4+FoxP3+ T-cell infiltration, extreme in comparison to the other DRESS cases, may have led to a somewhat abrogated clinical phenotype in this patient and meant that the clinicians felt it reasonable to try and treat through the reaction. A differential impact of topical steroid therapy may also have played a factor in explaining this picture.

Several other, non-Treg related, immune mechanisms may underpin DRESS in the context of progressive HIV. We have outlined several possible hypotheses and the data for them in a recent review article (5). Some of these factors include: increased number of immature pro-inflammatory dendritic cells in the context of progressive CD4 T-cell depletion (65); loss of the co-inhibitory mechanisms critical to the maintenance of peripheral tolerance [similar to that proposed to underline the increased drug hypersensitivity associated with checkpoint inhibition (66)]; and increased oxidative stress and micronutrient deficiencies accompanying HIV that lead to excessive ‘danger-signals’ that, augmented, limit drug-specific effector responses (67). Furthermore, similarities have been noted between the clinical features and state of aberrant immune activation with excess cytokine production between HIV-related immune-reconstitution syndrome and DRESS (68, 69).

## Limitations

This study was limited to sample size, particularly HIV-negative DRESS cases. The inability to rechallenge some patients (i.e., in the event of death) limited our ability for complete validation of some cases as single or multiple drug reactors, resulting in lower numbers in the comparative analysis. Sampling timeframes were based on clinical time-points and not strictly specified; therefore, some laboratory results, i.e., HIV viral loads or CD4 counts were unavailable to match with the timing of skin biopsy sampling. Thus, the lack of correlation between some of these values and skin biopsy findings may have been impacted.

## Conclusion

Although uncommon, SCARs remain a major clinical challenge for HIV/TB co-infected patients. This novel study uncovered some preliminary findings to guide further study. HIV-positive and negative DRESS was associated with an increased infiltration of cytotoxic CD8+ T-cells compared to normal skin, displaying their role as key mediators of tissue damage. CD4+ T-cells were lower in the skin of HIV-positive compared to HIV-negative DRESS patients, in line with the known HIV-related peripheral circulation decline in CD4 cell counts. While inter-individual variation was high, the frequency of dermal CD4+FoxP3+ T-cells was higher in HIV-positive DRESS patients reacting to more than one drug, and the functional significance of this requires further research. Additional work examining the role of skin TRM cells, as well as tissue macrophages and DCs in the context of HIV infection is now required.

## Data availability statement

The original contributions presented in the study are included in the article/Supplementary material, further inquiries can be directed to the corresponding author.

## Ethics statement

The studies involving human participants were reviewed and approved by University of Cape Town Faculty of Health Sciences Human Research Ethics Committee. The patients/participants provided their written informed consent to participate in this study.

## Author contributions

JP, RL, and MP developed the objectives and research questions. TC and RR conducted the experiments. TC, PC, SP, MP, RR, RL, and JP performed data analysis and interpretation. TC and JP wrote the manuscript and all authors participated in the revisions and approval of the final manuscript. All authors contributed to the article and approved the submitted version.

## Funding

The Immune-Mediated Adverse Drug Reactions-Africa project is part of the European and Developing Countries Clinical Trials Partnership 2 program supported by the European Union (Grant number TMA2017SF-1981). JP is supported by the National Institutes of Health Fogarty career development award (K43TW011178-04). PC is supported by the National Institutes of Health Fogarty PHD fellowship (D43 TW010559) and South African Medical Research Council (SAMRC) through its Division of Research Capacity Development under the Bongani Mayosi National Health Scholars Program. RL’s work is supported by the SAMRC and non-rated researcher support from the South African National Research Foundation (NRF). TC received financial support from the University of Cape Town Faculty of Health Sciences Department of Medicine Research Committee, and training in research that was supported by the Fogarty International Center of the National Institutes of Health and the Eunice Kennedy Shriver National Institute of Child Health & Human Development (NICHD) under Award Number D43 TW010559. The content is solely the responsibility of the authors and does not necessarily represent the official views of the National Institute of Health.

## Conflict of interest

The authors declare that the research was conducted in the absence of any commercial or financial relationships that could be construed as a potential conflict of interest.

## Publisher’s note

All claims expressed in this article are solely those of the authors and do not necessarily represent those of their affiliated organizations, or those of the publisher, the editors and the reviewers. Any product that may be evaluated in this article, or claim that may be made by its manufacturer, is not guaranteed or endorsed by the publisher.

## Abbreviations

ALT: alanine transferase
BSA: body surface area
CADR: cutaneous adverse drug reaction
cART: combinational antiretroviral therapy
DRESS: drug reaction with eosinophilia and systemic symptoms
ELISpot: enzyme-linked immunospot assay
FoxP3: forkhead box protein 3
FDC: fixed dose combination
FLTD: first-line anti-tuberculosis drug
IHC: immunohistochemistry
IMARI: immune mediated adverse drug reactions
HIV: human immunodeficiency virus
PLHIV: persons living with HIV
RegiSCAR: registry of severe cutaneous adverse reactions
SCAR: severe cutaneous adverse drug reactions
SDC: sequential drug rechallenge
TB: tuberculosis
TEN: toxic epidermal necrolysis
Treg: regulatory T-cell
