# Supplementary material for: Skin infiltrating T-cell profile of drug reaction with eosinophilia and systemic symptoms (DRESS) reactions among HIV-infected patients
Source: Front Med (Lausanne). 2023 May 5;10:1118527. doi: 10.3389/fmed.2023.1118527 (PMC10196146; doi:10.3389/fmed.2023.1118527)
Supplement: Supplementary file 1 [file Data_Sheet_1.docx]

**Supplementary Table S1**. Antibodies used for immunohistochemistry and immunofluorescence staining of skin tissue.

| Primary antibodies | | | | | | |
| --- | --- | --- | --- | --- | --- | --- |
| Panel | **Antibody (clone)** | **Supplier** | **Antigen Retrieval** | **Dilution in PBS** | **Incubation time** | **Positive control tissue** |
| Standard IHC | Rabbit anti-CD3 (SP7) | Abcam | Citrate | 1:150 | 1 hour | Tonsil |
|  | Rabbit anti-CD4 (EPR6855) | Abcam | Tris-EDTA | 1:400 | 1 hour |  |
|  | Rabbit anti-CD8 (Ab4055) | Abcam | Tris-EDTA | 1:250 | 1 hour |  |
|  | Mouse anti-CD45RO (UCHL1) | Biolegend | Tris-EDTA | 1:200 | 1 hour |  |
| CD3+CD4+FoxP3+ IF | Rat anti-CD3 (CD3-12) | Abcam | Tris-EDTA | 1:250 | Overnight | Appendix |
|  | Rabbit CD4 (EPR855) | Abcam | Tris-EDTA | 1:50 | Overnight |  |
|  | Mouse anti-FOXP3 (236A/E7) | Abcam | Tris-EDTA | 1:50 | Overnight |  |
| Secondary antibodies | | | | | | |
| Panel | **Antibody (clone)** | **Supplier** | **Antigen Retrieval** | **Dilution in PBS** | **Incubation time** | **Positive control tissue** |
| Standard IHC | Horseradish peroxidase anti-rabbit (K4003) | DAKO | - | Neat | 30mins | - |
|  | Horseradish peroxidase anti-rabbit (K4001) | DAKO | - | Neat | 30mins |  |
| CD3+CD4+FoxP3+ IF | Donkey anti-rat A488 | UCT confocal unit | - | 1:250 | 1 hour | - |
|  | Donkey anti-rabbit A647 | UCT confocal unit | - | 1:500 | 1 hour |  |
|  | Donkey anti-mouse Cy3 | UCT confocal unit | - | 1:1000 | 1 hour |  |
|  | DAPI | UCT confocal unit | - | 1:5000 | 10mins |  |

Abbreviations: DAPI, 4′.6-diamidino-2-phenylindole; EDTA, ethylenediaminetetraacetic acid; IHC, immunohistochemistry; IF, immunofluorescence; PBS, phosphate buffer saline; Tris, tris(hydroxymethyl)aminomethane; UCT, University of Cape Town

**Supplementary Table S2.** Description of type of reactions, laboratory findings and adjunct ELISpot and HLA data for HIV-positive DRESS patients.

| **Patient #** | **Age/Sex** | **CD4/**  **ART (Y/N) ^a^** | **Viral load (copies/mL)** | **Validated phenotype (RegiSCAR score)** | **Latency period (days)** | **Rash to biopsy (day) ^b^** | **Reaction characteristics at baseline and after drug rechallenge (timing) ^c^** | **Highest drug Naranjo score ^d^** | **Discharge regimen** | **Single or Multiple** | **Liver function tests & eosinophils** | **ELISpot SFU ^e^** | **HLA genotype** |
| --- | --- | --- | --- | --- | --- | --- | --- | --- | --- | --- | --- | --- | --- |
| 1 | 26/F | 66/Y | 59148 | Probable DRESS (5) | 43 | 8 | **Baseline: RHZE, Cotrimoxazole** – 30% BSA skin rash, exanthema, pain over affected area, with liver involvement.  **Rechallenge: INH (2 days)** – headache, abdominal pain, vomiting blood, elevated AST & ALT, hypotenuse, temperature 38°C. NOT STOPPED  **†MXF & ETA reaction**: abdominal pain, temperature spike, tender palms | PZA (4), EMB (4), Cotrimoxazole (4),  INH (3) | RIF, INH, TRD | Multiple | ALT (238), AST (304), ALP (173), GGT (156), Eosinophils (0.5) | INH: 130  EMB: 62  TMP/SMX§: 0 | A 68:27:01G + 74:01:01G  B 07:05:01G + 35:01:01G  C 04:01:01G + 07:02:01G |
| 2 | 22/F | 384/Y | LDL | Definite DRESS (6) | 29 | 5 | **Baseline**: **Tribuss, Trivuda, INH** – 60% BSA skin rash involving the eyes, lip, and oral mucosa, exanthema, erythema, burning, nausea, muscle pain, sore throat, facial oedema (*Not rechallenged*) | INH (4),  NVP (4) ‡ | TDF, FTC, EFV (ART FDC) | Undetermined | ALT (227), AST (84),  ALP (268), GGT (708), Eosinophils (0.57) | INH: 0 | A 26:01:01G + 33:03:07G  B 44:03:01G + 44:03:02G  C 02:10:01G + 07:01:01G |
| 3 | 30/F | 48/Y | 17778 | Definite DRESS (7) | 28 | 5 | **Baseline: RHZE, Cotrimoxazole** – 80% BSA skin rash, exanthema, peeling (desquamation), and erythema, with liver involvement, facial oedema (*Not rechallenged*) | Cotrimoxazole (4) ‡ | RIF, INH, EMB, PZA | Single | ALT (237), AST (136), ALP (81),  GGT (78), Eosinophils (1.52) | TMP/SMX§: 5 | A 23:01:01G + 68:02:01G  B 15:03:01G + 53:01:01G  C 0:10:01G + 04:01:01G |
| 4 | 45/M | 39/Y | 93907 | Definite DRESS (6) | 8 | 11 | **Baseline: RHZE** – 80% BSA skin rash, erythema, itching, facial oedema, liver involvement  **Rechallenge: PZA (within 24hrs)** – itching, visible rash, fever, exanthema, erythema | PZA (6) | RIF, INH, EMB | Single | ALT (75),  AST (137), ALP (120), GGT (140), Eosinophils (7.44) | PZA: 13  INH: 40 | A 24:02:01G + 34:02:01G  B 07:02:01G + 08:01:01G  C 07:01:01G + 07:02:01G |
| 5 | 38/F | 87/N | LDL | Possible DRESS (2) ● | 7 | 23 | **Baseline: RHZE** – 75% BSA skin rash involving oral and genital mucosa, exanthema, itching, burning (*Not rechallenged*) | RIF (4) ‡ | INH, PZA, EMB, Rfb | Single | ALT (92),  AST (58),  ALP (110), GGT (91),  Eosinophils (0.14) | RIF: 13  Rifabutin: 6 | A 30:01:01G + 30:02:01G  B 14:02:01G + 42:01:01G  C 08:02:01G + 17:01:01G |
| 6 | 26/F | 330/N | 262654 | Definite DRESS (7) | 25 | 4 | **Baseline: RHZE** – 65% BSA skin rash, exanthema, and erythema, itching, burning, pain over affected area, facial oedema  **Rechallenge: PZA (48hrs)** – itching, visible rash, exanthema, erythema | PZA (6) | RIF, INH, EMB | Single | ALT (49), AST (54), ALP (88), GGT (36),  Eosinophils (2.91) | PZA: 0 | A 32:01:01G + 68:01:01G  B 58:01:01G + 58:02:01G  C 06:02:01G + 06:02:01G |
| 7 | 41/F | 39/N | 3475 | Definite DRESS (6) | 32 | 7 | **Baseline: RHZE** – 55% BSA skin rash, exanthema, and erythema, itching and burning  **Rechallenge: RIF (5 days)** – itching, burning, visible rash, exanthema, erythema.  **Rechallenge: Rifabutin (5 days)** – eosinophilia, visible rash, erythema | RIF (6), Rifabutin (6) | INH, EMB, PZA | Single | ALT (68), AST (119), ALP (111), GGT (187), Eosinophils (1.49) | RIF: 60 | A 02:01:01G + 68:02:01G  B 15:10:01G + 44:03:02G  C 03:04:02G + 07:01:01G |
| 8 | 30/F | 137/N | 115989 | Possible DRESS (3) ● | 20 | 5 | **Baseline: RHZE**, Cotrimoxazole – 65% BSA skin rash involving the lip and oral mucosa, peeling (desquamation), erosions, erythema itching, burning, cough  **Rechallenge: INH (72hrs)** – visible rash, erythema, fever, nausea | INH (6) | PZA, EMB, MXF, Rfb | Single | ALT (13),  AST (22),  ALP (83),  GGT (22),  Eosinophils (1.04) | INH: 3 | A 01:01:01G + 68:02:01G  B 57:02:01G + 58:01:01G  C 07:01:01G + 07:01:01G |
| 9 | 45/F | 66/N | - | Probable DRESS (5) | 55 | 5 | **Baseline: RHZE**, **Cotrimoxazole**: 40% BSA skin rash involving the scalp, erythema, itching, peeling (desquamation), facial oedema, vomiting, anorexia, abdominal pain (*Not rechallenged*) | RHZE (3), Cotrimoxazole (4) ‡ | Deceased | Single  (Positive cotrimoxazole ELISpot) | ALT (35),  AST (60),  ALP (61),  GGT (138),  Eosinophils (0.68) | RIF, PZA, INH: 0  EMB: 25  4-NIT-10: 45  4-NIT-100: 5 | A 02:01:01G + 34:02:01G  B 15:03:01G + 45:01:01G  C 02:10:01G + 16:01:01G |
| 10 | 47/F | 578/N | 3862114 | Definite DRESS (7) | 10 | 9 | **Baseline: RHZE** – 70% BSA skin rash involving the scalp, lips, oral and nasal mucosa, erythema, burning, fatigue, anorexia, sore throat, kidney, and liver involvement  **Rechallenge: PZA (24hrs)** – abdominal pain, elevated AST and ALT, erythema | PZA (6) | RIF, INH, EMB, MXF | Single | ALT (183), AST (90),  ALP (82),  GGT (134),  Eosinophils (0.19) | PZA: 0 | A: *alleles not typed*  B 15:03:01G + 15:03:01G  C 02:10:01G + 04:01:01G |
| 11 | 39/F | 360/Y | - | Possible DRESS (3) ● | 14 | 22 | **Baseline: RHZE** – 80% BSA skin rash involving oral mucosa, erythema, itching peeling (desquamation), nausea, joint and abdominal pain, cough, liver involvement  **Rechallenge: RIF (3 days)** – worsening rash | RIF (6) | Deceased | Single | ALT (56),  AST (190), ALP (790), GGT (749), Eosinophils (0.21) | PZA: 0 RIF:0  INH: 0  TMP/SMX§/#: 0  4-NIT10/100: 0 | Not done |
| 12* | 45/M | 141/Y | - | Definite DRESS/EN overlap (6) | 35 | 9 | **Baseline: RHZE, Cotrimoxazole** – 65% BSA skin rash involving the nails, lips, oral and nasal mucosa, erythema, peeling (desquamation), erosions, liver, and lung involvement*  **Rechallenge: INH with EMB and Rifabutin (10 days)** – erythema, blisters, fever, peeling, epidermal necrosis (Possible TEN). | INH (6), EMB (6), Rfb (6) | BDQ, LZD, LVX, CFZ | Multiple | ALT (37),  AST (63),  ALP (70),  GGT (102),  Eosinophils (0.13) | EMB, PZA: 0  INH: 7  4-NIT-10: 52  TMP/SMX§: 2 | A 29:02:01G + 68:02:01G  B 44:03:02G + 53:01:01G  C 04:01:01G + 07:01:01G |
| 13 | 54/F | 86/N | - | Probable DRESS (5) | 27 | 23 | **Baseline: RHZE, Cotrimoxazole** – 60% BSA skin rash involving the scalp, erythema, itching, burning, fatigue, headache, fever, enlarged lymph nodes. | Cotrimoxazole (4) | RIF, INH, PZA, EMB | Single  (Some cotrimoxazole ELISpot signal) | ALT (23),  AST (27),  ALP (71),  GGT (46),  Eosinophils (0.04) | TMP/SMX§ = 10 | A 02:02:01G + 29:02:01G  B 42:01:01G + 58:02:01G  C 06:02:01G + 17:01:01G |
| 14 | 41/M | 162/Y | - | Probable DRESS (4) | 11 | 14 | **Baseline: RHZE** – 20% BSA skin rash, erythema, itching, infiltration, liver involvement  **Rechallenge: RIF & EMB together** – pruritis, induration, liver derangement | RIF (6),  EMB (6) | BDQ, LZD, CFZ, TZD, LVX | Undetermined | ALT (31),  AST (63),  ALP (392), GGT (122), Eosinophils (1.21) | RIF, INH, PZA, EMB: 0  TMP/SMX§: 0 | A 03:01:01G + 29:02:01G  B 08:01:01G + 15:03:01G  C 02:10:01G + 07:02:01G |
| 15§ | 35/M | 142/Y | LDL | Definite DRESS (7) | 70 | 73 | **Baseline: RHZE** – 80% BSA skin rash involving the eye, lip, oral and nasal mucosa, erythema, peeling (desquamation), facial oedema, liver involvement  **†Rifinah reaction (unclear timing):** deranged liver functions  **†RIF, INH reaction (non-FDC) (unclear timing):** deranged liver functions | RIF (6),  INH (6) | RIF, INH⁋ | Multiple | ALT (35),  AST (48),  ALP (456), GGT (307), Eosinophils (0.37) | RIF; INH: 0 | A 30:02:01G + 36:01:01G  B 53:01:01G + 53:01:01G C 04:01:01G + 04:01:01G |

^a^ CD4 count (cells/mm^3^)/on ART at time of sampling (yes/no). ^b^ Time from disease onset to biopsy, days. ^c^ Drug rechallenged (time from drug rechallenge to positive reaction). ^d^ Naranjo scoring (Scores: ≥9 (Definite), 5 to 8 (Probable), 1 to 4 (Possible)). ^e^ Positive ELISpot ≥50 spot forming units (SFU) per million cells.

**†** Not rechallenged to drug but had a separate CADR. **‡**Patient not rechallenged to drug(s) – highest Naranjo/Alden scored most likely offending drug(s). ⁋ later reacted to individual RIF & INH and pushed through. ● Retrospective “Possible” cases of DRESS included by expert opinion of two dermatologists. * Skin biopsy sampled at baseline where patient initially presented with DRESS. § Patient 15 excluded from all analyses and detailed as a unique case report.

ELISpot drug concentrations: RIF (25µg/ml), INH (50µg/ml), PZA (50µg/ml), EMB (50µg/ml), Rifabutin (25µg/ml), TMP/SMX (50/250µg/ml§ or 500/2500µg/ml#), 4-NIT-10 (10µg/ml) 4-NIT-100 (100µg/ml), Vancomycin (250 µg/ml).

Abbreviations: ALT, alanine transaminase; ALP, alkaline phosphatase; ART, antiretroviral therapy; BSA, body surface area; EFV, efavirenz; ELISpot, enzyme-linked immunosorbent spot; EN, epidermal necrolysis; ETA, ethionamide; FDC, fixed dose combination; FTC, emtricitabine; GGT, gamma-glutamyl transferase; HLA, human leukocyte antigen; LDL, lower than detectable limit; LVX, levofloxacin; MXF , moxifloxacin; NVP, nevirapine; Rfb, rifabutin; RHZE, rifampicin/isoniazid/pyrazinamide/ethambutol FDC; TMP/SMX, trimethoprim/sulfamethoxazole; TDF, tenofovir; TRD, terizidone; 4-NIT-10, 4-nitro sulfamethoxazole-10 (TMP/SMX metabolite).

**Supplementary Table S3.** Description of type of reactions, laboratory findings and adjunct ELISpot and HLA data for HIV-negative DRESS patients.

| Patient# | Age/Sex | Validated phenotype (RegiSCAR score) | Latency period (days) | Rash to biopsy (days) ^a^ | Reaction characteristics at baseline, and after drug rechallenge (timing) ^b^ | Highest drug Naranjo score ^c^ | Discharge regimen | Single or Multiple | Liver function tests and eosinophils | Elispot SFU ^d^ | HLA genotype |
| --- | --- | --- | --- | --- | --- | --- | --- | --- | --- | --- | --- |
| 1 | 15/M | Definite DRESS (**6**) | 34 | 3 | **Baseline: Phenytoin –** 40% BSA, pustules and erythema, fatigue, headache, anorexia, nausea, liver involvement. (*Not rechallenged*) | Phenytoin (7) | Deceased | Single | ALT (94), AST (239), ALP (281), GGT (139),  Eosinophils (0.62) | Not done | A: *alleles not typed*  B 27:09 + 39:10:01G  C 01:02:01G + 12:03:01G |
| 2 | 57/F | Definite DRESS (**6**) | 23 | 17 | **Baseline: Phenytoin –** 90% BSA skin rash involving the scalp, lips, liver, and oral, nasal, and genital mucosa, peeling (desquamation), erythema, facial oedema (*Not rechallenged*) | Phenytoin (7) | Nil | Single | ALT (220), AST (327), ALP (95), GGT (602),  Eosinophils (6.09) | Not done | A 30:01:01G + 66:01:01G  B 42:02:01G + 58:02:01G  C 06:02:01G + 17:01:01G |
| 3 | 52/F | Probable DRESS (**5**) | 69 | 3 | **Baseline: Carbamazepine –** 40% BSA skin rash involving the eyes, erythema, itching, fever, liver, and lung involvement. (*Not rechallenged*) | Carbamazepine (7) | LEV | Single | ALT (564), AST (83), ALP (798), GGT (655),  Eosinophils (1.36) | Not done | Not done |
| 4 | 15/F | Probable DRESS (**5**) | 32 | 6 | **Baseline: RHZE –** 40% BSA skin rash involving the oral mucosa, erythema, facial oedema, itching, fatigue, nausea, abdominal pain, fever, cough, liver involvement.  **Rechallenge**: **INH (14hrs)** – visible rash, itching, purpura, erythema, fever.  **Rechallenge: RIF (8.5hrs)** – fever, burning eyes, morbilliform rash, palmar erythema, elevated AST & ALT | RIF (6), INH (6), EMB (6) | CFZ, BDQ, LZD, LVX | Multiple | ALT (159), AST (197), ALP (430), GGT (106), Eosinophils (1.36) | RIF: 520  EMB: 17  PZA: 0  INH: 33 | A 25:01:01G + 32:01:01G  B 07:02:01G + 15:01:01G  C 02:10:01G + 03:03:01G |
| 5 | 46/M | Definite DRESS (**7**) | 36 | 8 | **Baseline: Allopurinol –** 40% BSA skin rash, erythema, facial oedema, purpura, itching, burning, fever.  †**Vancomycin rection** – visible rash, abnormal pain, jaundice, weakness of limbs | Allopurinol (7) | Nil | Multiple | ALT (67), AST (38), ALP (105), GGT (131), Eosinophils (2.55) | Not done | A 34:02:01G + 66:01:01G  B 44:03:01G + 57:03:01G  C 04:02:01G + 18:01:01G |

^a^ Time from disease onset to biopsy, days. ^b^ Drug rechallenged (time from drug rechallenge to positive reaction). ^c^ Naranjo scoring (Scores: ≥9 (Definite), 5 to 8 (Probable), 1 to 4 (Possible)). ^d^ Positive ELISpot ≥50 spot forming units (SFU) per million cells.

ELISpot drug concentrations: RIF (25µg/ml), INH (50µg/ml), PZA (50µg/ml), EMB (50µg/ml).

Abbreviations: ALT, alanine transaminase; ALP, alkaline phosphatase; BDQ, bedaquiline; BSA, body surface area; CFZ, clofazimine; ELISpot, enzyme-linked immunosorbent spot; EMB, ethambutol; FDC, fixed dose combination; GGT, gamma-glutamyl transferase; HLA, human leukocyte antigen; INH, isoniazid; LEV, levetiracetam; LVX, levofloxacin; LZD, linezolid; PZA, pyrazinamide; RHZE, rifampicin/isoniazid/pyrazinamide/ethambutol FDC; RIF, rifampicin.

**Supplementary Table S4.** Summary of clinical and laboratory data for HIV-positive and negative normal skin controls

| Control# | Age/Sex | HIV Status | CD4 count (cells/ mm^3^) | Eosinophil count  (x10^9^/L cells) | Reason and type of surgical procedure |
| --- | --- | --- | --- | --- | --- |
| 1 | 43/Female | Positive | 508 | 0.11 | Breast carcinoma, bilateral mastectomy |
| 2 | 35/Female | Positive | 513 | 0.04 | Breast reduction |
| 3^a^ | 39/Female | Positive | Unknown | Unknown | Fibroadenoma, unilateral breast reduction |
| 4 | 83/Female | Negative | - | 0.16 | Breast carcinoma, bilateral mastectomy |
| 5 | 36/Female | Negative | - | 0.03 | Breast carcinoma, bilateral mastectomy |
| 6 | 52/Female | Negative | - | 0.02 | Excess stomach skin used for breast reconstruction |

^a^CD4 and eosinophil counts data could not be retrieved for this control. For patients with non-invasive cancers undergoing breast mastectomies, normal non-cancerous skin was obtained at a site away from the tumour in the case of unilateral mastectomies, or from the non-cancerous breast in the case of bilateral mastectomies.

Abbreviations: HIV, human immunodeficiency virus.

**Supplementary Table S5.** Quantification of dermal and epidermal T-cell infiltrates amongst HIV-positive and negative DRESS and normal skin

|  |  | **Average number of positive cells per high-powered field** | | | | | |
| --- | --- | --- | --- | --- | --- | --- | --- |
|  |  | **HIV-positive DRESS (n=14)** | **HIV-positive normal skin (n=3)** | **p-value** | **HIV-negative DRESS (n=5)** | **HIV-negative normal skin (n=3)** | **p-value** |
| **EPIDERMIS:** | CD3 | 4 (2 - 10) | 0 (0 - 2) | 0.11 | 10 (8 - 11) | 0 (0 - 2) | **<0.001** |
|  | CD4 | 1 (0 - 2) | 0 (0 - 2) | 0.17 | 6 (5 - 8) | 0 (0 - 0) | **0.02** |
|  | CD8 | 2 (1 - 5) | 0 (0 - 0) | **0.02** | 6 (3 - 7) | 0 (0 - 0) | **0.02** |
|  | CD45RO | 2 (1 - 8) | 0 (0 - 0.3) | **0.01** | 4.3 (3 - 7) | 0 (0 - 2) | 0.11 |
|  | CD4/CD8 | 0.3 (0.0 - 0.5) | 0 | **0.16** | 1.7 (1.2 – 2.8) | 0 | **0.03** |
|  |  |  |  |  |  |  |  |
| **DERMIS:** | CD3 | 53 (45 - 62) | 23 (22 - 24) | **0.02** | 81 (63 - 106) | 25 (24 - 27) | **<0.01** |
|  | CD4 | 19 (14 - 24) | 19 (18 - 20) | 1.00 | 64 (46 - 99) | 15 (14 - 18) | **0.03** |
|  | CD8 | 37 (30 - 51) | 11 (10 - 12) | **0.003** | 50 (27 - 87) | 6 (5 - 7) | **<0.001** |
|  | CD45RO | 42 (33 - 50) | 18 (16 - 19) | **0.02** | 55 (36.8 - 81.0) | 22 (21 - 24) | **0.01** |
|  | CD4/CD8 | 0.4 (0.3 – 0.6) | 1.9 (1.6 – 2.0) | **0.002** | 1.8 (1.2 – 2.2) | 2.5 (2.1 – 3.4) | 0.26 |

Cell counts are median (IQR). The Wilcoxon rank sum test was used to determine statistical significance between groups. P values < 0.05 were considered significant, recorded in **bold.**


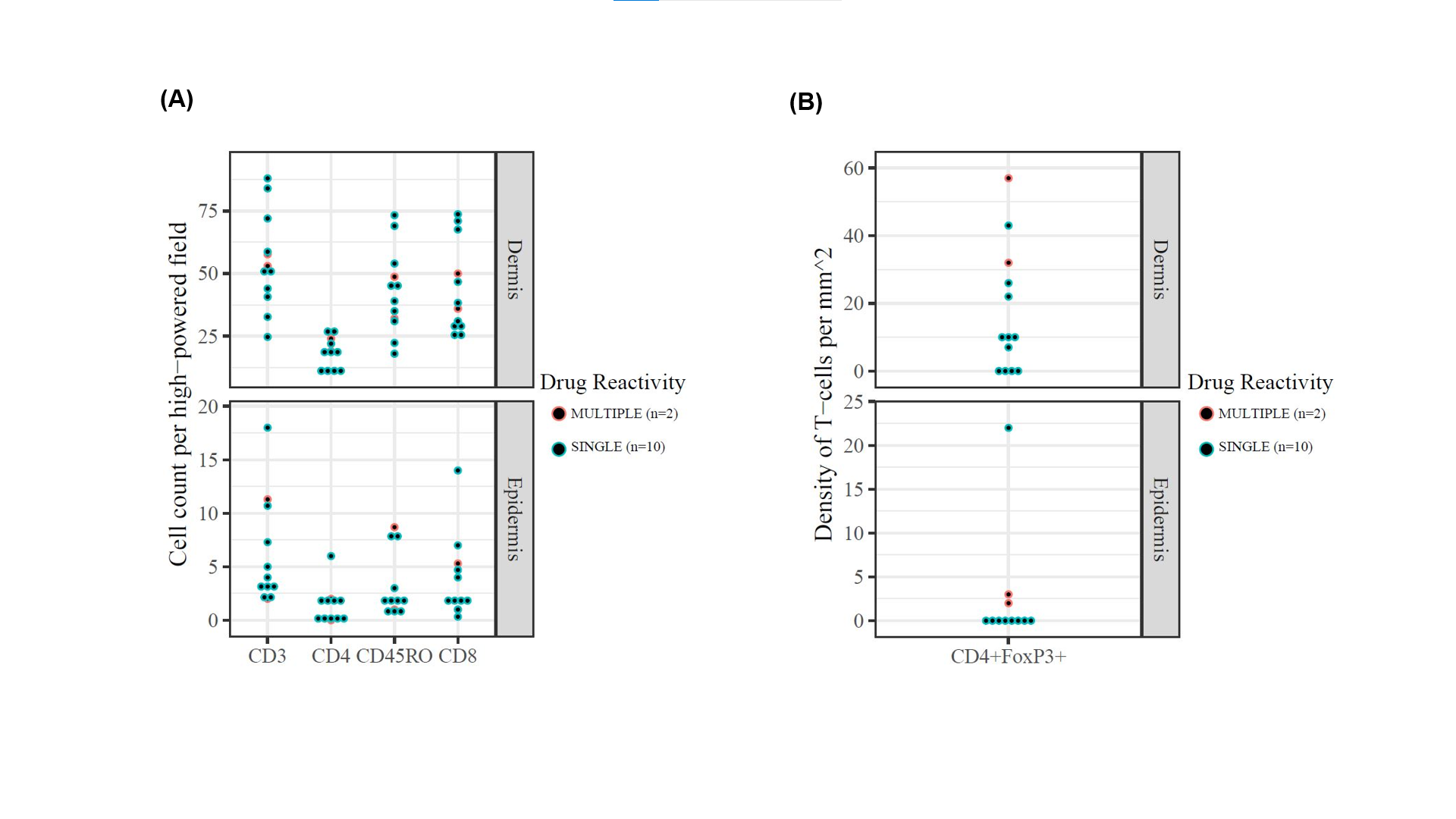


**Supplementary Figure S1.** Comparison of dermal and epidermal T-cell infiltrates amongst HIV-positive DRESS single and multiple drug reactors. Immunohistochemistry quantification of the different T cell infiltrates (A) and immunofluorescence quantification of CD4+FoxP3+ T cells (B) amongst HIV-positive DRESS cases.

**
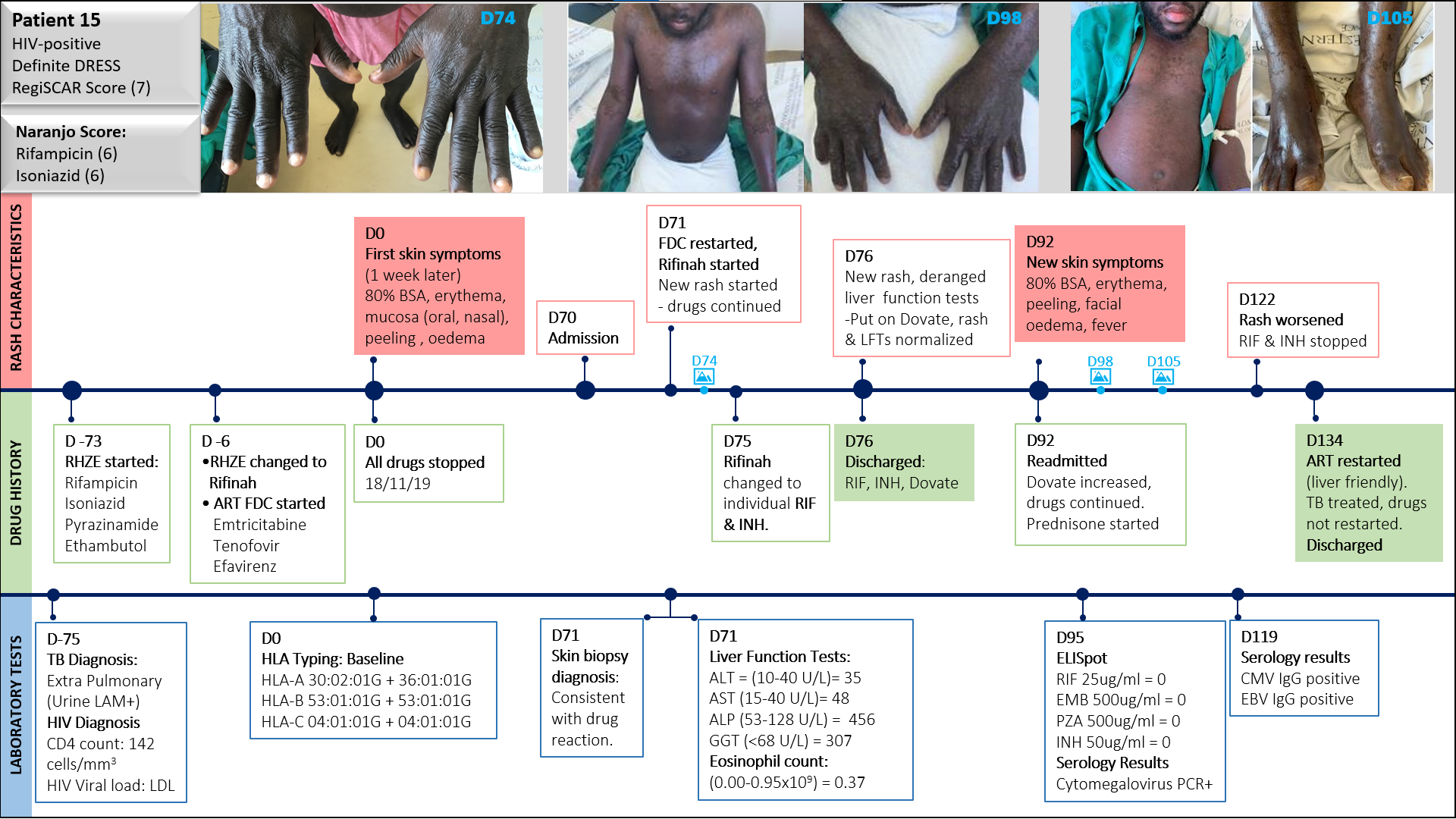
Supplementary Figure S2.** Disease progression timeline for patient 15, an HIV-positive definite DRESS case sampled 73 days after onset of clinical symptoms. Abbreviations: ALT, alanine transaminase; ALP, alkaline phosphatase; ART, antiretroviral therapy, AST, aspartate transaminase; BSA, body surface area; CMV, cytomegalovirus; DRESS, drug reaction with eosinophilia and systemic symptoms; EBV, Epstein-Barr virus; Eos, eosinophils; ELISpot, enzyme-linked immunosorbent spot; EMB, ethambutol; FDC, fixed dose combination, GGT, gamma-glutamyl transferase; GSH, Groote Schuur hospital; HIV, human immunodeficiency virus; HLA, human leukocyte antigen; IgG, immunoglobulin G; INH, isoniazid; LAM, lipoarabinomannan; LDL, lower than detectable limit; PCR, polymerase chain reaction; PZA, pyrazinamide; RHZE, rifampicin/isoniazid/pyrazinamide/ethambutol FDC; RIF, rifampicin; TB, tuberculosis.

**Supplementary Table S6.** Association between density of dermal CD4+ and CD4+FoxP3+ T-cells and clinical/demographic characteristics in HIV-positive and negative DRESS patients.

| INDEPENDENT VARIABLE | Dependent variable r^2^ (coefficient, p value) | | | |
| --- | --- | --- | --- | --- |
|  | **HIV-positive DRESS (n=14)** | | **HIV-negative DRESS (n=5)** | |
|  | **Dermal CD4+** | **Dermal** **CD4+FoxP3+** | **Dermal CD4+** | **Dermal** CD**4+FoxP3+** |
| Age | 0.256 (-0.506, 0.065) | **0.402 (-0.634, 0.015)** | 0.304 (-0.551, 0.336) | 0.494 (0.703, 0.189) |
| Days (symptoms to onset) | 0.00 (-0.016, 0.957) | 0.028 (-0.168, 0.567) | 0.141 (-0.375, 0.534) | **0.869 (0.932, 0.021)** |
| Serum CD4 count | 0.039 (0.191, 0.514) | 0.05 (0.223, 0.444) | - | - |
| Log viral load ^a^ | 0.143 (-0.379, 0.315) | 0.136 (-0.368, 0.33) | - | - |
| Eosinophils | 0.223 (-0.472, 0.089) | 0.06 (-0.244, 0.401) | 0.163 (-0.403, 0.501) | **0.945 (0.972, 0.006)** |
| Liver enzymes: AST | 0.095 (0.308, 0.284) | 0.258 (0.508, 0.064) | 0.071 (-0.267, 0.664) | 0.465 (0.682, 0.205) |
| ALP | 0.00 (0.006, 0.983) | 0.011 (-0.104, 0.723) | 0.037 (-0.192, 0.757) | 0.314 (-0.560, 0.326) |
| ALT | **0.289 (0.538, 0.047)** | **0.441 (0.664, 0.01)** | 0.006 (-0.081, 0.897) | 0.018 (-0.133, 0.831) |
| GGT | 0.031 (0.176, 0.547) | 0.013 (0.112, 0.715) | 0.146 (-0.382, 0.525) | 0.304 (0.552, 0.335) |
| Body surface area of rash | 0.00 (-0.02, 0.946) | 0.00 (-0.018, 0.951) | 0.046 (-0.215, 0.729) | **0.909 (0.954, 0.012)** |
| RegiSCAR severity score | 0.013 (-0.114, 0.697) | 0.019 (0.139, 0.636) | 0.051 (0.227, 0.714) | 0.138 (-0.371, 0.538) |

^a^ HIV viral loads were log transformed to permit the use of a linear model. P values ≤ 0.05 were considered statistically significant, recorded in **bold.**

Abbreviations: ALT, alanine transaminase; ALP, alkaline phosphatase; AST, aspartate transaminase; DRESS, drug reaction with eosinophilia and systemic symptoms; GGT, gamma-glutamyl transferase; HIV, human immunodeficiency virus; RegiSCAR, registry of severe cutaneous adverse drug reactions.
